# The auxin-inducible degradation (AID) system enables versatile conditional protein depletion in *C. elegans*

**DOI:** 10.1242/dev.129635

**Published:** 2015-12-15

**Authors:** Liangyu Zhang, Jordan D. Ward, Ze Cheng, Abby F. Dernburg

**Affiliations:** 1 Department of Molecular and Cell Biology, University of California, Berkeley, Berkeley, CA 94720-3220, USA; 2 Howard Hughes Medical Institute, 4000 Jones Bridge Road, Chevy Chase, MD 20815, USA; 3 Life Sciences Division, Department of Genome Dynamics, Lawrence Berkeley National Laboratory, Berkeley, CA 94720, USA; 4 California Institute for Quantitative Biosciences, Berkeley, CA 94720, USA; 5 Department of Cellular and Molecular Pharmacology, University of California, San Francisco, CA 94158, USA

**Keywords:** *C. elegans*, Genetic tool, Degron, Auxin, Auxin-inducible degradation, Tissue-specific depletion

## Abstract

Experimental manipulation of protein abundance in living cells or organisms is an essential strategy for investigation of biological regulatory mechanisms. Whereas powerful techniques for protein expression have been developed in *Caenorhabditis elegans*, existing tools for conditional disruption of protein function are far more limited. To address this, we have adapted the auxin-inducible degradation (AID) system discovered in plants to enable conditional protein depletion in *C. elegans*. We report that expression of a modified *Arabidopsis* TIR1 F-box protein mediates robust auxin-dependent depletion of degron-tagged targets. We document the effectiveness of this system for depletion of nuclear and cytoplasmic proteins in diverse somatic and germline tissues throughout development. Target proteins were depleted in as little as 20-30 min, and their expression could be re-established upon auxin removal. We have engineered strains expressing TIR1 under the control of various promoter and 3′ UTR sequences to drive tissue-specific or temporally regulated expression. The degron tag can be efficiently introduced by CRISPR/Cas9-based genome editing. We have harnessed this system to explore the roles of dynamically expressed nuclear hormone receptors in molting, and to analyze meiosis-specific roles for proteins required for germ line proliferation. Together, our results demonstrate that the AID system provides a powerful new tool for spatiotemporal regulation and analysis of protein function in a metazoan model organism.

## Abstract (precis)

**Summary:** The auxin-inducible degradation (AID) system is adapted to *C. elegans* to enable conditional depletion of degron-tagged protein targets in as little as twenty minutes.

## INTRODUCTION

Techniques for precise temporal and spatial control of protein expression enable detailed analysis of developmental mechanisms. In *Caenorhabditis elegans*, a variety of tools for stage- or tissue-specific expression have been developed, including the *hsf-1* system (Bacaj and Shaham, 2007), drug-induced protein stabilization (Cho et al., 2013), FLP-mediated excision of FRT-flanked transcriptional terminators (Davis et al., 2008), and the Q-system (Wei et al., 2012). However, available methods for conditional protein depletion are far more limited. Depletion of gene products in specific stages and tissues has been achieved through RNAi (Qadota et al., 2007), or by gene disruption via tissue-specific expression of sequence-specific nucleases (Cheng et al., 2013; Shen et al., 2014). However, these approaches are indirect and irreversible, as they rely on inactivation of a gene or on mRNA degradation. Additionally, there is often a substantial lag between induction and protein depletion, the duration of which depends on mRNA and/or protein stability (Elbashir et al., 2001; Fire et al., 1998).

Degrons, amino acid sequences that direct proteasomal destruction of tagged proteins, have become extremely powerful experimental tools, particularly in yeast. A recent report repurposed an endogenous, developmentally regulated degradation pathway in *C. elegans* (Armenti et al., 2014) for experimental manipulation of proteins in this system. In cells or tissues engineered to express ZIF-1, an E3 ubiquitin ligase substrate-recognition subunit, proteins fused to a 36 amino acid degron, a zinc finger domain from the PIE-1 protein (ZF1), can be quickly degraded. This system holds great promise, but also has some limitations. It cannot be used in the germ line, as the native role of this pathway is to degrade germline-expressed proteins upon fertilization, and ectopic ZIF-1 expression would therefore disrupt essential germline functions. Conditional depletion using this system also relies on *zif-1* induction by heat shock, which can interfere with some processes and requires some lag time.

The auxin-inducible degradation (AID) system of plants has enabled rapid, conditional protein depletion in yeast and cultured vertebrate cells (Holland et al., 2012; Nishimura et al., 2009). This system relies on expression of a plant-specific F-box protein, TIR1, which regulates diverse aspects of plant growth and morphogenesis in response to the phytohormone auxin (Gray et al., 1999; Ruegger et al., 1998). TIR1 is the substrate recognition component of a Skp1–Cullin–F-box (SCF) E3 ubiquitin ligase complex, which recognizes substrates only in the presence of auxin (indole-3-acetic acid, or IAA) and targets them for degradation by the proteasome (Dharmasiri et al., 2005; Kepinski and Leyser, 2005; Tan et al., 2007). When expressed in heterologous systems, TIR1 can interact with endogenous Skp1 and Cullin proteins to form a functional, auxin-dependent ubiquitin E3 ligase (Holland et al., 2012; Kanke et al., 2011; Kreidenweiss et al., 2013; Nishimura et al., 2009; Philip and Waters, 2015). However, to our knowledge, this approach has not been previously used in any intact metazoan system.

We have now adapted the AID system for small-molecule inducible protein degradation in *C. elegans* (Fig. S1A). We report that expression of TIR1 enables rapid, reversible, auxin-dependent degradation of nuclear and cytoplasmic targets in all tissues and developmental stages tested. We have applied this system to analyze control of molting by nuclear hormone receptors and meiosis-specific roles for proteins required for germline proliferation, demonstrating the versatility of this system for dissecting protein function in a widely used model organism.

## RESULTS

### Design strategy for the auxin-inducible degradation (AID) system in *C. elegans*

TIR1-dependent protein degradation has been most extensively characterized in the model plant *Arabidopsis thaliana.* The TIR1 gene from rice (*Oryza sativa*) was found to yield more robust degradation when expressed in budding yeast and vertebrate cells than the orthologs from *Arabidopsis* and *Gossypium hirsutum* (cotton) (Nishimura et al., 2009). However, the standard laboratory culture temperature for *C. elegans* (20°C) is closer to the preferred range for *Arabidopsis* (23-25°C), so we chose to express the *Arabidopsis* TIR1 protein sequence in *C. elegans*, in part to take advantage of prior molecular studies of this protein. We constructed a synthetic TIR1 gene that was codon optimized for *C. elegans* and contains two introns (Fig. S1B). We incorporated two point mutations (D170E and M473L) shown to increase the affinity of AtTIR1 for its substrates and to increase auxin sensitivity without causing auxin-independent activity (Yu et al., 2013) (Fig. S1B,C). This gene was fused to a codon-optimized red fluorescent protein (mRuby) gene (Rog and Dernburg, 2015) to permit visualization of TIR1 expression, and placed under the control of several different germline and somatic regulatory elements (Fig. S1D). Throughout this study, the *unc-54* 3′ UTR was used for all somatic TIR1 drivers, and the *sun-1* 3′ UTR was used for germline expression. Worm strains with integrated copies of these transgenes were created by transposon-mediated single-copy insertion (MosSCI) (Frokjaer-Jensen et al., 2008).

We fused a 44-amino acid minimal degron sequence (Morawska and Ulrich, 2013) (Fig. S1E,F) derived from the *Arabidopsis thaliana* IAA17 protein to two broadly expressed *C. elegans* genes, *smu-2* and *dhc-1*, together with a synthetic GFP gene (Rog and Dernburg, 2015) to enable visualization and monitoring of the target proteins (Fig. S1G). The splicing regulator SMU-2 was selected as a candidate nuclear target because it is one of two known genes that can be readily expressed from extrachromosomal arrays in both the soma and the germ line (Spartz et al., 2004), facilitating strain construction. We engineered a *degron::smu-2::GFP* transgene and confirmed robust protein expression from extrachromosomal arrays. The dynein heavy chain (DHC-1) was chosen as a cytoplasmic target because it is ubiquitously expressed and essential, and because of our interest in the roles of dynein during meiotic prophase (Sato et al., 2009; Wynne et al., 2012). A degron-GFP tag was inserted at the 3′ end of the endogenous *dhc-1* coding sequence using CRISPR/Cas9-mediated editing (Dickinson et al., 2013). We also constructed strains expressing degron-tagged GFP from a stably inserted transgene, which served as a functionally inert reporter (Fig. S1G).

### The AID system degrades cytoplasmic and nuclear proteins at all developmental stages

**Fig. 1.**
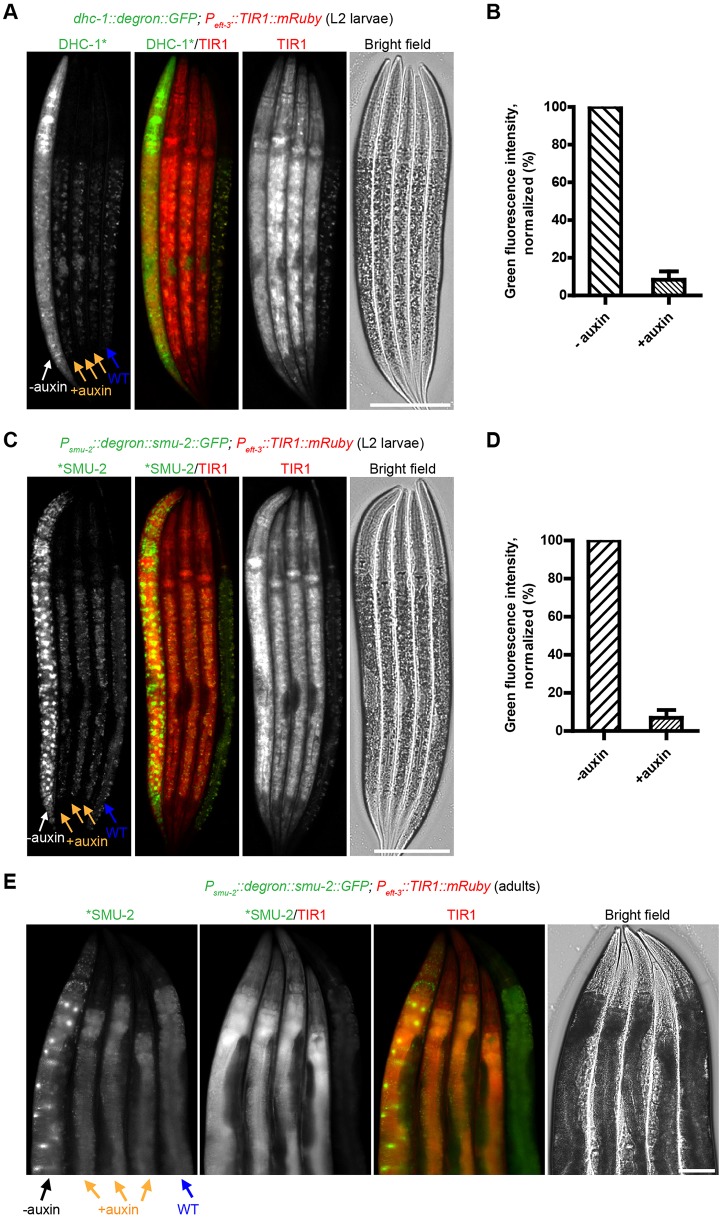
**The auxin-inducible degradation (AID) system enables degradation of cytoplasmic and nuclear proteins in larval and adult *C. elegans*.** (A) Inducible degradation of the cytoplasmic dynein heavy chain, DHC-1, in the soma. Animals with a degron-GFP cassette inserted at the 3′ end of the endogenous *dhc-1* gene in a *P_eft-3_::TIR1::mRuby::unc-54* 3′ UTR genetic background were treated with (+) or without (−) 1 mM auxin for two hours. Worms were then immobilized and imaged. Wild-type (WT) worms treated with 1 mM auxin for two hours were included to measure background fluorescence. L2 larvae are shown for clarity because their germ line has not yet proliferated extensively, facilitating observation of somatic degradation; other developmental stages are shown in Fig. S2. (B) Quantification of DHC-1-degron-GFP degradation in A. Data are presented as the mean±s.d. from three independent experiments (*n*=18 worms). (C) Inducible degradation of nuclear SMU-2 protein in the soma. L2 larvae expressing degron-SMU-2-GFP from an extrachromosomal array and mRuby-tagged TIR1 from an integrated transgene were treated, immobilized and imaged as described in A. (D) Quantification of degron-SMU-2-GFP degradation in C. Data are presented as the mean±s.d. from three independent experiments (*n*=17 worms). (E) Inducible degradation in the adult soma. Young adult worms expressing degron-SMU-2-GFP and TIR1-mRuby were treated with (+) or without (−) 1 mM auxin for three hours and then immobilized and imaged as described in A. Scale bars: 50 μm.

We first introduced a construct designed to express TIR1-mRuby from the strong, ubiquitous *eft-3* (also known as *eef-1A.1*) promoter and the 3′ UTR from *unc-54*, which is broadly permissive for somatic expression. Visualization of red fluorescence confirmed that TIR1 was expressed in most or all somatic tissues throughout development, but not in the germline, as expected. We crossed this transgene to a strain in which the endogenous *dhc-1* gene was C-terminally tagged with a degron-GFP cassette using CRISPR/Cas9-mediated editing. In the presence of 1 mM auxin, DHC-1 was depleted within two hours in somatic tissues at various developmental stages, from L1 to adult (Fig. 1A,B; Fig. S2). Degradation was quantified in L2 larvae by determining green fluorescence intensity in whole worms, which showed a 91.6% reduction in GFP signal in auxin treated animals compared with control animals. The residual fluorescence is likely due to undegraded DHC-1 in the germ line, where TIR1 was not expressed, as the somatic fluorescence was no higher than in wild-type animals lacking GFP-tagged *dhc-1*.

We also crossed extrachromosomal arrays encoding a degron- and GFP-tagged SMU-2 protein into our pan-somatic TIR1 strain to test whether this nuclear protein could be targeted by the TIR1 ubiquitin ligase complex. When these animals were exposed to 1 mM auxin, fluorescent SMU-2 disappeared throughout the soma within two hours in L2 larvae and three hours in adult worms (Fig. 1C-E). Thus, the AID system permits protein depletion throughout larval and adult stages.

**Fig. 2.**
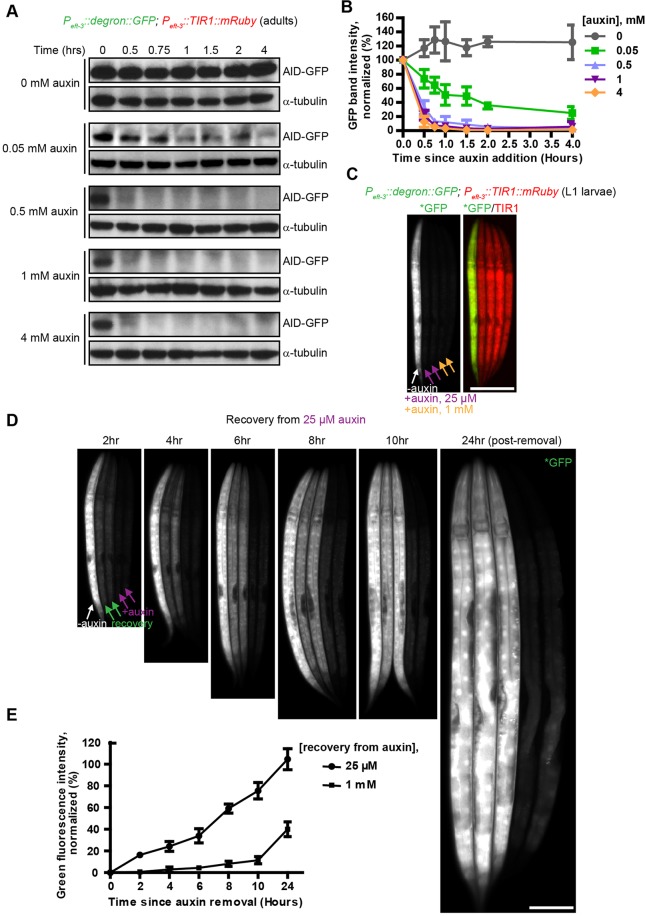
**AID-mediated degradation is rapid and reversible.** (A) Young adult worms expressing degron-tagged GFP and TIR1-mRuby from the same somatic driver (*P_eft-3_; unc-54* 3′ UTR) were treated with auxin in S basal buffer containing OP50. Worms were then lysed at various time points, and western blots were performed using antibodies against GFP and tubulin. (B) Degradation rates were determined using the blots shown in A. Data are presented as mean±s.d. from three independent experiments. (C) Low concentrations of auxin permit efficient degradation in larvae. L1 larvae expressing degron-GFP and TIR1-mRuby were treated with 25 μM or 1 mM auxin (+) or without (−) auxin for two hours. Worms were then immobilized and imaged as described in Fig. 1. (D) Conditional degradation is reversible following removal of auxin. L1 larvae treated with 25 μM auxin for two hours in C were transferred onto fresh NGM plates. Recovery of degron-tagged GFP was examined at the indicated time points. Worms without auxin treatment and those left on auxin plates were included as controls. (E) Quantification of the relative recovery rates in D (recovery from 25 μM auxin) and in Fig. S3 (recovery from 1 mM auxin). Data are presented as means±s.d. from three independent experiments. Scale bars: 50 μm.

To analyze the kinetics of AID-mediated protein degradation in adult animals, we used a strain in which TIR1-mRuby and degron-tagged GFP were co-expressed using the same regulatory sequences (the *eft-3* promoter and *unc-54* 3′ UTR). The abundance of degron-tagged GFP was measured over time in adult worms exposed to a range of auxin concentrations. We found that the degradation rate depended on the concentration of auxin: in the presence of ≥0.5 mM auxin, degron-GFP was reduced to 50% of its initial level within 20 min, and was undetectable within 45 min (Fig. 2A,B), whereas lower auxin concentrations resulted in slower depletion of degron-GFP.

### AID-mediated degradation is reversible

We next investigated whether AID-mediated protein degradation is reversible. We treated L1 larvae with various concentrations of auxin, and monitored the depletion and recovery of degron-tagged GFP using fluorescence imaging. We found that treatment with 25 μM auxin was sufficient to eliminate degron-GFP within two hours (Fig. 2C). The recovery rate depended strongly on the concentration of auxin used for depletion (Fig. 2C-E, Fig. S3): after removal from 25 μM auxin, visible GFP fluorescence was observed within two hours, and reached half the level seen in untreated animals within seven hours (Fig. 2D,E). When degradation was induced with 1 mM auxin, recovery of GFP expression required substantially longer (Fig. 2E, Fig. S3). Thus, auxin concentrations should be tested and optimized for specific targets and tissues, particularly for experiments in which reversibility is desired.

We note that rates of AID-mediated protein degradation and recovery also depended on the developmental stage of the treated animals (data not shown): degradation occurred more quickly in young larvae than in adults. This might reflect differences in the rates of auxin uptake or diffusion through tissues, as well as potential differences in the abundance of expressed TIR1 and other endogenous SCF components at various developmental stages. The recovery rate of specific proteins will clearly depend on transcription and translation rates, which are also likely to vary during development.

### The AID system enables functional analysis of nuclear hormone receptors during development

**Fig. 3.**
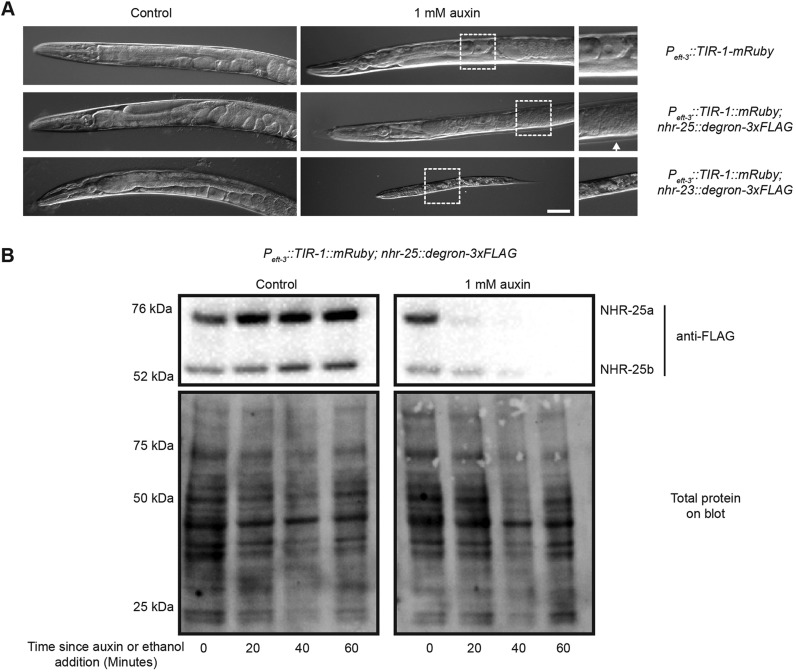
**The AID system enables functional analysis of nuclear hormone receptors during development.** (A) Representative images of animals from the timed egg lay on control or 1 mM auxin plates following 60 h at 25°C. In the absence of auxin, no defects were seen in any genotype; however, in the presence of auxin, worms expressing degron-tagged NHR-25 and pan-somatic TIR1 showed molting defects (arrow indicated unshed cuticle) and gonadal defects such as tumorous germ lines (note lack of eggs and abnormal germ line). Animals expressing degron-tagged NHR-23 and pan-somatic TIR1 uniformly arrested as L1 larvae. Scale bar: 50 µm. (B) Temporal analysis of inducible protein degradation. Worms of the indicated genotypes were grown for six hours at 25°C following dauer release before 0.25% ethanol (control) or 1 mM auxin were added and samples harvested every 20 min. Lysates were resolved by SDS-PAGE and immunoblotted with the indicated antibodies. Stain-free (Bio-Rad) analysis of total protein on each blot is provided as a loading control. Two isoforms of NHR-25 (a and b) are detected, as previously described (Ward, 2015).

Because of our interest in nuclear hormone receptor-mediated control of developmental gene regulatory networks (Ward et al., 2013, 2014), as test cases, we targeted two essential nuclear hormone receptors, NHR-23 and NHR-25. A *degron::3xFLAG* cassette was PCR amplified with 79-85 bp homology arms (Paix et al., 2014) and inserted into the 3′ ends of the endogenous *nhr-23* and *nhr-25* coding sequences using *pha-1* co-conversion (Ward, 2015). This approach enabled homozygous knock-in animals to be obtained within nine days of injection. These alleles were then crossed into the pan-somatic *P_eft-3_::TIR1::mRuby* strain. In the absence of auxin, the resulting strains showed normal brood sizes and viability, demonstrating that the tags did not interfere with NHR-23 or NHR-25 function (Table 1). However, following treatment with 1 mM auxin, the *nhr-25* degron line displayed a strong reduction in brood size (Table 1), and a spectrum of other defects consistent with inactivation of NHR-25 (Asahina et al., 2006; Chen et al., 2004): gonad abnormalities and molting defects (Fig. 3A, Table 1), and complete sterility among the F1 progeny of treated animals. Auxin treatment of the degron-tagged *nhr-23* animals did not affect the number of their F1 progeny (Table 1), but 100% of these progeny arrested as L1 larvae, which were also dumpy (Fig. 3A, Table 1). In a previous study, when *nhr-23* was inactivated by RNAi at the same stage (L4) at which we initiated auxin treatment, only 2% of progeny arrested at the L1 stage, with additional progeny arresting at L2 and L3 (Kostrouchova et al., 2001). These data indicate that the AID system can produce more penetrant phenotypes than depletion by RNAi.

**Table 1.**
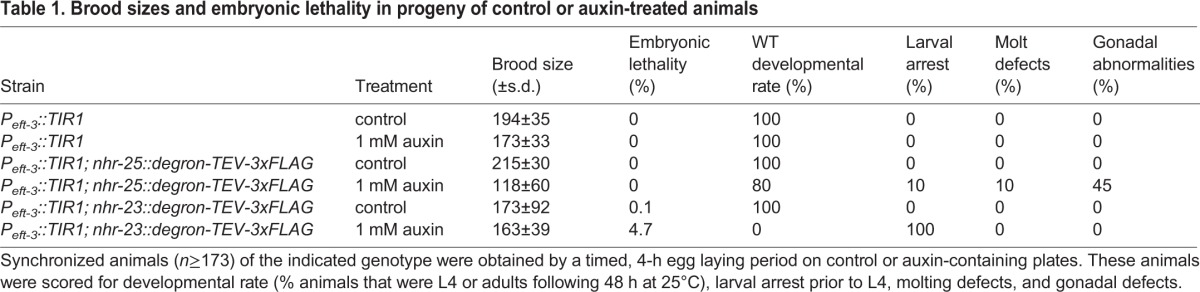
**Brood sizes and embryonic lethality in progeny of control or auxin-treated animals**

We next wished to assess the kinetics of depletion of these nuclear hormone receptors. A time course of NHR-25-degron-3xFLAG expression following dauer release revealed that expression increased dramatically over the next 6-8 h (Fig. S4), so we focused on this time window. We released animals from dauer arrest in liquid culture by feeding with HB101 bacteria for six hours. Animals were then treated with either 0.25% ethanol (control) or 1 mM auxin, and NHR-25-degron-3xFLAG levels were monitored by anti-FLAG immunoblotting. Whereas NHR-25-degron-3xFLAG levels were unchanged in the control, the target was largely depleted within 20 min after auxin addition, and almost undetectable after 40 min (Fig. 3B). Thus, this approach enables precise time-resolved analysis of proteins that are dynamically expressed during development. This versatility should also allow modulation of protein levels in large cultures, permitting new types of biochemical experiments.

### The AID system allows tissue-specific protein depletion

**Fig. 4.**
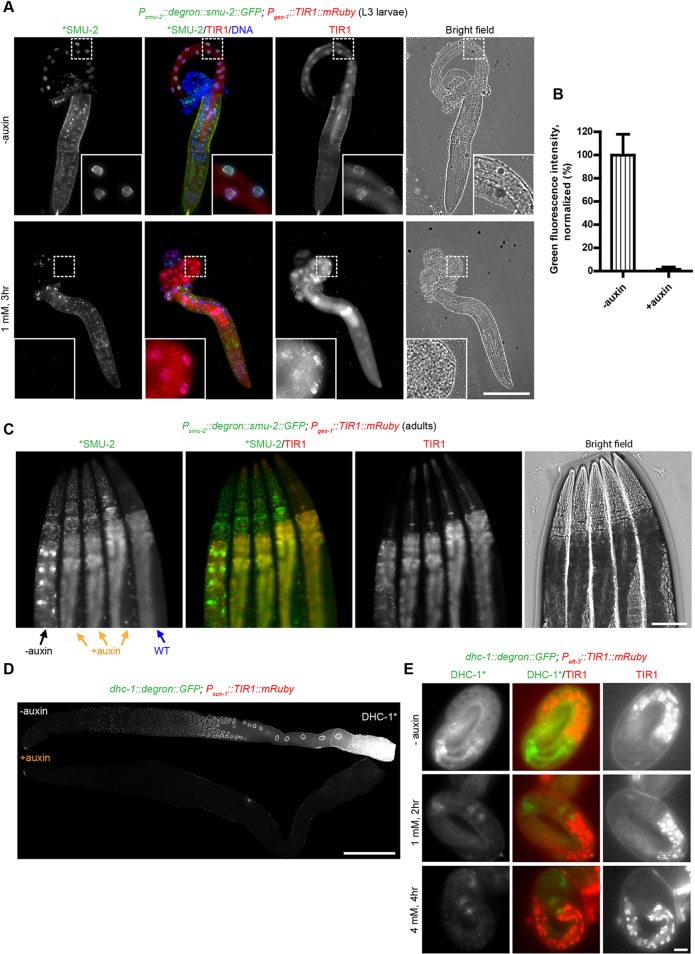
**The AID system permits tissue-specific degradation in *C. elegans*.** (A) The *ges-1* promoter was used to drive TIR1 expression in the intestine. L3 larvae carrying this transgene and degron-tagged SMU-2 from arrays were treated with (+) or without (−) 1 mM auxin for three hours. Worms were then dissected and intestines were extruded to monitor residual SMU-2-GFP in this tissue. DNA was stained with DAPI to indicate the nuclei. Insets show higher-magnification views of the outlined regions. (B) Quantification of degron-SMU-2-GFP degradation in the intestine. Data are presented as the mean±s.d. from three independent experiments (*n*=144 nuclei, 15 worms). (C) Tissue-specific degradation in adults. Young adult worms expressing degron-SMU-2-GFP from arrays and TIR1 in the intestine were treated with (+) or without (−) 1 mM auxin for three hours. Wild-type worms (WT) treated with auxin were included as background control. (D) Inducible degradation in the germ line. Young adults expressing TIR1 driven by the *sun-1* promoter and 3′ UTR along with degron-tagged DHC-1 were treated with (+) or without (−) 1 mM auxin for two hours. Worms were then dissected, fixed, and imaged. (E) Inducible degradation in embryos. Eggs laid by hermaphrodites expressing *dhc-1::degron::GFP* and *P_eft-3_::TIR1::mRuby::unc-54* 3′ UTR were treated with 1 mM or 4 mM auxin (+) or without (−) auxin in S basal buffer for indicated times. Scale bars: 50 µm in A,C,D; 5 μm in E.

We next tested whether target proteolysis could be spatially restricted to specific somatic tissues. We generated a strain expressing a *TIR1-mRuby* transgene under control of a *myo-2* promoter. After confirming that red fluorescence was specifically detected in pharyngeal muscle, we crossed this strain to one expressing the ubiquitously expressed *dhc-1::degron::GFP* transgene. After exposure to 1 mM auxin, we observed an obvious decline in green fluorescence within the pharynx, whereas DHC-1-degron-GFP in other tissues remained unchanged relative to untreated controls (Fig. S5A). Restriction of TIR1 expression to the intestine by driving it from the *ges-1* promoter resulted in loss of DHC-1-degron-GFP only in the gut (Fig. S5B). Similarly, we observed auxin-dependent depletion of the degron-tagged nuclear protein SMU-2 only in tissues where TIR1 was expressed (Fig. 4A-C). To quantify the tissue-specific depletion, we dissected worms specifically expressing TIR1 in the intestine and determined the depletion. of degron-SMU-2-GFP in intestine by measuring the green fluorescence in intestinal nuclei. We determined that 98.3% of SMU-2 was degraded in intestine following auxin treatment (Fig. 4B).

One of our key goals in developing the AID system was to enable conditional protein depletion in the germ line. To express TIR1 throughout this tissue, we used the promoter and 3′ UTR from the *sun-1* gene, which is expressed in both the mitotic and meiotic regions of the germ line as well as in mitotic cells of the early embryo (Malone et al., 2003; Minn et al., 2009). When this transgene was crossed into worms expressing degron-tagged DHC-1-GFP, we observed the disappearance of DHC-1 fluorescence throughout the germ line, including in maturing oocytes, where expression was initially strongest (Fig. S6A). We confirmed that tagged DHC-1 was undetectable throughout the germ line by dissection following auxin exposure (Fig. 4D). Notably, DHC-1 was undetectable in the early embryos inside the mothers following auxin treatment (Fig. S6A). Thus, the *P_sun-1_::TIR1::mRuby* trangene is an effective tool for depleting target proteins in early embryos as well as throughout the mitotic and meiotic germ line.

Because *C. elegans* embryos are surrounded by an eggshell and vitelline membrane that prevent entry of many molecules (Carvalho et al., 2011), we considered it unlikely that the AID system would be useful in embryos after they were laid. Nevertheless, we tested the system by treating embryos expressing TIR1 and degron-tagged DHC-1-GFP with auxin. To avoid weakening the eggshell by treatment with bleach, we allowed adult hermaphrodites to lay embryos on auxin-free plates, then washed these embryos into S basal medium containing 1 mM or 4 mM auxin. Unexpectedly, we observed a striking reduction in GFP fluorescence following auxin treatment. Although depletion of the target was incomplete (Fig. S6B), high magnification revealed that the residual green fluorescence was spatially restricted to cells in which little or no TIR1-mRuby was expressed from our *P_eft-3_*/*unc-54* 3′ UTR transgene (Fig. 4E). These observations indicate that auxin can penetrate the eggshell and induce effective target proteolysis in embryos. Development of other drivers for TIR1 would likely enable auxin-mediated proteolysis in specific embryonic tissues.

We conclude that the AID system provides a robust tool for conditional depletion of proteins of interest in many, and likely all, somatic and germline tissues.

### Conditional degradation of DHC-1 in the germ line reveals multiple roles in meiotic progression

**Fig. 5.**
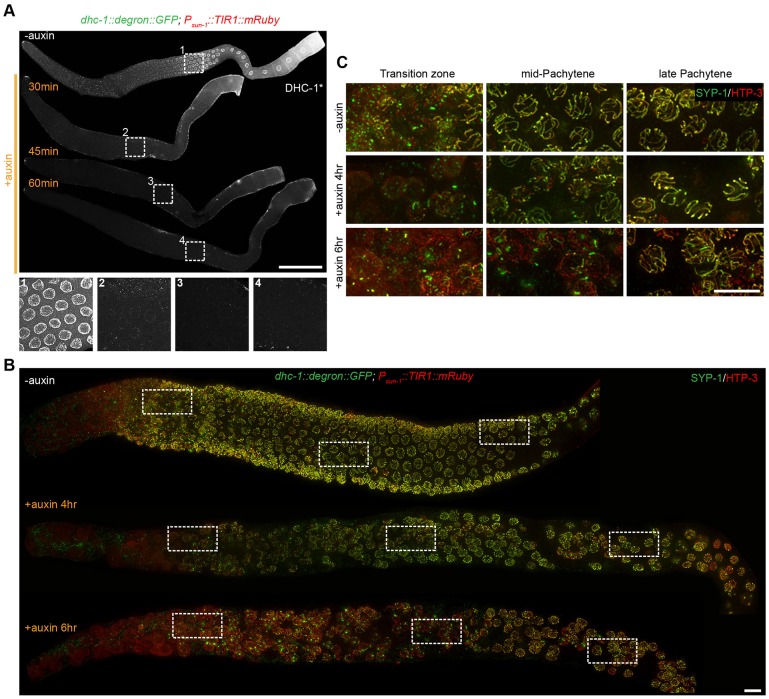
**Conditional depletion of DHC-1 in the germ line reveals its essential function in meiosis.** (A) Rapid degradation of DHC-1-degron-GFP in the germ line. *dhc-1::degron::GFP*; *P_sun-1_::TIR1::mRuby* young adult animals were treated with 1 mM auxin (+) or without (−) auxin for the indicated time. Worms were then dissected, fixed, stained, and imaged. Four enlarged images are included to indicate efficient degradation. (B) Low-magnification views of germ lines stained for SYP-1 (green) and HTP-3 (red) to monitor synapsis. SYP-1 is a synaptonemal complex protein, whereas HTP-3 is a component of the chromosome axes (MacQueen et al., 2002, 2005). Synapsis defect was indicated by mislocalization of SYP-1. *dhc-1::degron::GFP*; *P_sun-1_::TIR1::mRuby* adults were treated with (+) or without (−) 1 mM auxin for the indicated times. (C) Higher magnification views from the corresponding regions in B. Scale bars: 50 μm in A; 5 μm in B,C.

To validate the utility of the AID system to interrogate germline functions, we analyzed the effects of depleting the dynein heavy chain protein (DHC-1). Meiosis is a specialized cell division process characterized by homologous chromosome pairing, synapsis, and segregation into daughter cells. Using RNAi and temperature sensitive mutants, dynein activity was previously demonstrated to be essential for formation of the synaptonemal complex during *C. elegans* meiosis (Sato et al., 2009), which in turn is required for stable lengthwise association between homologous chromosomes. These earlier experiments were complicated by the essential role of dynein in mitotic chromosome segregation, which is required for germline proliferation. We first exposed animals expressing GFP- and degron-tagged DHC-1 and our pan-germline TIR1 (*P_sun-1_::TIR1::mRuby)* to 1 mM auxin to monitor the degradation kinetics of DHC-1 by fluorescence. We found that DHC-1 became undetectable throughout the germ line within 45 min of auxin treatment, and verified this by immunofluorescence (Fig. 5A).

To determine the effects of DHC-1 depletion on meiotic prophase, we dissected animals after exposure to auxin for several hours, so that a pool of nuclei had entered and progressed through early prophase in the absence of DHC-1. Early meiotic nuclei in these germ lines showed obvious defects in chromosome synapsis, as indicated by aberrant localization of SYP-1 (Fig. 5B,C), a synaptonemal complex protein (MacQueen et al., 2002), consistent with our previous findings (Sato et al., 2009). We also observed effects that had not been apparent when dynein was depleted by RNAi or temperature-sensitive mutations. These included global disorganization of germline nuclei (Fig. S7), consistent with a previously demonstrated role of dynein in maintaining nuclear position in germline cells (Zhou et al., 2009), as well as a novel defect in oocyte maturation (Fig. S7). These effects likely reflect a more complete abrogation of dynein function than we were able to achieve by RNAi and/or temperature shifts.

### Viability, fertility and development are unaffected by TIR1 expression and auxin treatment

The utility of any technique in addressing biological mechanisms relies on it having minimal off-target effects. So far, we have observed no obvious side effects of either long-term auxin exposure within the useful concentration range or from TIR1 expression. We found that expression of TIR1 from strong drivers in the soma or germ line had no effect on brood size or embryonic viability at either 25°C (Table 1) or 20°C (Table S1). Because we were concerned that expression of TIR1 might sequester other SCF complex components, we specifically looked for phenotypes associated with inactivation of the germline-expressed Skp1-related proteins (SKR-1 and SKR-2) (Nayak et al., 2002) in the germ lines of animals expressing TIR1 from the strong *sun-1* promoter, but observed no such abnormalities. Moreover, neither exposure to 1 mM auxin nor TIR1 expression affected developmental rate (Table S2). We did observe a modest reduction in brood size when animals were exposed to 4 mM auxin for extended periods at 25°C (Table S3). This might be an indirect effect, as bacterial growth was somewhat inhibited at this concentration. Because the maximal rate of target degradation is obtained at lower concentrations (Fig. 2B), auxin should be used at 1 mM or lower concentrations, conditions under which we observed no apparent side effects.

## DISCUSSION

Rapid, conditional and reversible protein depletion is an invaluable tool for probing protein function in cellular or developmental processes. A variety of methods have been developed to conditionally stabilize or destabilize proteins (Armenti et al., 2014; Banaszynski et al., 2006; Bonger et al., 2011; Caussinus et al., 2012; Cho et al., 2013; Dohmen et al., 1994; Raina and Crews, 2010; Renicke et al., 2013; Taxis et al., 2009; Zhou et al., 2000), but many of these approaches are unsuitable for use in *C. elegans* or have significant limitations. Here, we demonstrate that the AID system allows efficient, rapid degradation of nuclear and cytoplasmic proteins at all developmental stages. Protein expression recovered upon auxin removal, with lower auxin doses facilitating faster recovery. By spatially restricting TIR1 expression through various promoter and 3′ UTR sequences, we achieved tissue-specific depletion of degron-tagged target proteins, with auxin exposure providing temporal control. Auxin concentration and the stage of the animal or embryo influence the degradation and recovery rates of target proteins. Accordingly, the relevant kinetic parameters might need to be tested and optimized for specific experiments. The developmental stage likely influences the rate of auxin uptake and diffusion or transport through tissues, the abundance of endogenous Skp1 and Cullin orthologs or other TIR1 partners, and the expression levels of both TIR1 and the target protein. For experiments in which recovery of expression is desired, it will also be important to consider gene-specific transcription and translation rates.

A number of features make the AID system particularly attractive and simple to implement. Auxin is inexpensive and is moderately water-soluble, making it easy to administer in plates or liquid growth media. Liquid culture is particularly well suited for experiments that require large amounts of starting material (e.g. IP-mass spectrometry, ChIP-seq). Whereas many drugs show poor efficacy in *C. elegans* because of limited permeability of the egg or cuticle, efficient export, and other toxin-resistance mechanisms (Broeks et al., 1995; Lindblom et al., 2001), we have found that exogenous treatment with auxin can induce target degradation at all developmental stages, even during embryogenesis. Auxin-mediated depletion is also efficient in the absence of food (data not shown) making it useful for analysis of processes induced by starvation, such as autophagy, L1 arrest or dauer formation. This feature should also allow production of large populations of synchronized animals depleted for a protein of interest. The small size of the degron enables efficient knock-in by co-conversion (Arribere et al., 2014; Kim et al., 2014; Ward, 2015), or selection-based CRISPR editing (Dickinson et al., 2015, 2013; Norris et al., 2015). The degron can be fused to the N- or C-terminus of target proteins, and can even be inserted internally. By fusing an epitope tag or fluorescent protein to the target along with the degron, the same engineered protein can be localized, purified, and inducibly degraded, providing a multifunctional tool for experimental biology. Crucially, exposure to auxin over the effective concentration range had no detectable effects on worm viability, morphology or fertility, nor did expression of TIR1 under strong drivers in the soma or germ line (Table 1, Tables S1-S3). These attributes should make the system applicable to a wide range of questions in cell and developmental biology.

Although the auxin-inducible degradation system we describe here is robust and specific, future directed optimization of the AID system might enhance its utility in *C. elegans*. We have characterized the system using a gain-of-function allele of the TIR1 gene from *Arabidopsis*, but it might be useful to compare the performance of this protein to orthologs or paralogs from other plants, with and without the corresponding mutations. Ongoing efforts to develop more potent auxin agonists might also provide other small molecule tools, although the high water solubility, small size, low toxicity, and nominal cost of auxin might prove difficult to improve upon. It might also be possible to develop smaller and/or higher-activity degron sequences or to enhance TIR1 activity through further evolutionary or mutational analysis. However, in its present incarnation, the AID system represents a highly versatile tool for rapid, conditional, robust tissue-specific and stage-specific protein degradation in *C. elegans*.

Provided that a model organism has a set of tissue-specific regulatory elements and that auxin can be delivered to a tissue of interest, our adaptation of the AID system for *C. elegans* should provide a road map for importing this technology into other metazoan model organisms.

## MATERIALS AND METHODS

### Constructs and generation of transgenic lines

Constructs used in this study are listed in Table S4. More information about constructs and transgenic lines are provided in supplementary materials and methods. The constructs and transgenic worm lines used in this study will be made available through AddGene and CGC, respectively.

### Strains

All strains were maintained on NGM plates at 20°C except where otherwise noted. Strains used in this study are listed in Table S5.

To obtain highly synchronized larvae without bleaching, adults of the indicated genotypes were transferred onto seeded NGM plates and allowed to lay eggs for 1 hour. Adults were then removed and the embryos were cultured for appropriate times to allow them to reach the indicated developmental stages. Synchronized adults were obtained by picking L4 larvae and maintaining them for 20-24 h at 20°C.

To obtain the synchronized L1 larvae used to generate the data in Table S2, two plates of each strain were suspended using M9 buffer. After a wash with M9 buffer, worms were bleached for 4 min. Eggs were then washed twice with M9 buffer, and starved in M9 buffer overnight to synchronize to L1 stage.

### Auxin treatment

Unless otherwise indicated, auxin treatment was performed by transferring worms to bacteria-seeded plates containing auxin. The natural auxin indole-3-acetic acid (IAA) was purchased from Alfa Aesar (#A10556). A 400 mM stock solution in ethanol was prepared and was stored at 4°C for up to one month. Auxin was diluted into the NGM agar, cooled to about 50°C, before pouring plates. Because we found that high concentrations of auxin (e.g. 4 mM) inhibited bacterial growth, a fresh OP50 culture was highly concentrated before spreading plates. Plates were left at room temperature for 1-2 days to allow bacterial lawn growth.

For auxin treatment in liquid culture, S basal buffer was supplemented with 3% (v/v) pelleted OP50 and the indicated concentration of auxin. For all auxin treatments, 0.25% ethanol was used as a control.

### Viability and fertility

To score total progeny (brood size) and male self-progeny, L4 hermaphrodites were picked onto individual plates with or without auxin, and transferred to new plates daily over 4 days. The eggs laid on each plate were counted after removing the parent. Viable progeny and male progeny were quantified when the F1 reached L4 or adult stages (2-3 days post egg laying).

### Microscopy and image acquisition

To permit direct comparisons of worms of different genotypes or experimental conditions, animals were lined up side-by-side on agarose pads immediately prior to imaging. Briefly, 2-3 µl of buffer containing 100 mM sodium azide was spotted on a freshly made 2% agarose pad, and 4-6 worms were then transferred into the liquid spot using a pick. As the liquid absorbed into the pad, worms were quickly manipulated to lie side-by-side, and overlaid with a coverslip. Fluorescence images were acquired immediately to avoid dehydration of the animals. Wide-field optical sections at 1-µm *z*-spacing were acquired with a DeltaVision Elite microscope (Applied Precision) using a 10× N.A. 0.40 air objective, and pseudocolored using the SoftWoRx package or Adobe Photoshop. Images were not deconvolved. For each data stack, a single optical section near the middle of the animals with the highest GFP signal was selected for presentation. For the images in Fig. 3A, animals were picked into 2-3 µl of buffer containing 100 mM sodium azide on a freshly made 2% agarose pad, and overlaid with a coverslip. Images were acquired using DIC optics and a 63× objective on an Axioplan 2 (Zeiss) microscope running Micromanager.

To quantify the degradation in Fig. 1B and D, all treatments and image collection were performed in parallel. Images were acquired as described above using a constant exposure for GFP (DHC-1-degron-GFP or degron-SMU-2-GFP), which was set to maximize signal-to-noise while avoiding camera saturation. Fluorescence quantification was performed on a single, unprocessed optical section from the middle of each data stack. Worms were outlined using the selection tool in ImageJ (National Institutes of Heath), and the average green fluorescence intensity for each animal was measured using a plugin (‘Analyze’-‘Measure RGB’) in ImageJ. Background intensity values, measured in wild-type worms treated in parallel with auxin, were subtracted from each measurement. The fluorescence intensity in treated worms was normalized by dividing the value for each worm by the measured intensity in an untreated worm in the same microscope field. An analogous approach was used to measure the rate of recovery of protein expression after auxin removal in Fig. 2E, with background intensities measured in worms that remained on auxin plates during the recovery period.

To quantify intestine-specific degradation of SMU-2, as reported in Fig. 4B, worms were dissected to extrude their intestines in 1× Egg Buffer (25 mM HEPES pH 7.4, 118 mM NaCl, 48 mM KCl, 2 mM EDTA, 0.5 mM EGTA) without detergents, then fixed with 1% formaldehyde for 2 min, washed with PBST, stained with DAPI, washed again, and mounted in glycerol-NPG mounting medium. Images were collected as stacks of 16 optical sections at intervals of 0.5 μm using a DeltaVision Elite microscope (Applied Precision) with a 20× N.A. 0.75 air objective. A maximum-intensity projection through the data stack was calculated. Individual intestinal nuclei in these images were manually segmented in ImageJ based on the DAPI signal, and their average green fluorescence intensity was measured as described above. Background fluorescence was measured in nuclei from wild-type worms treated in parallel, and this value was subtracted from the mean nuclear intensity value for each worm. These background-corrected values were expressed as a percent of the mean nuclear fluorescence intensity measured in control (non-auxin-treated) worms. Data were analyzed by Student's *t*-test and reported as mean±s.d. for all worms in three independent experiments.

Immunofluorescence experiments were performed according to published protocols (Phillips et al., 2009). Briefly, young adult hermaphrodites (20-24 h post-L4) were dissected in Egg Buffer containing 15 mM sodium azide and 0.1% Tween 20, followed by fixation with 1% formaldehyde in the same buffer on a coverslip for 1 min. The coverslip with worms was then picked up using a Histobond slide (VWR), blotted to remove any excess fixative, and frozen on dry ice. After removal of the coverslip, slides with adhered worms were transferred to −20°C methanol for 1 min. Samples were then washed in PBST (PBS containing 0.1% Tween 20) and blocked with Blocking Reagent (Roche) in PBST. Primary antibody incubations were performed overnight at 4°C. After washing with PBST, secondary antibody incubations and DAPI staining were conducted sequentially at room temperature. Primary antibodies used were as follows: guinea pig anti-HTP-3 (1:500; MacQueen et al., 2005), rabbit anti-SYP-1 (1:500; MacQueen et al., 2002). Secondary antibodies labeled with Cy3 or Cy5 were purchased from Jackson ImmunoResearch (1:500, #106-165-003 or #111-175-144). All images were acquired as *z*-stacks through 8-µm depth at intervals of 0.2 μm using a DeltaVision Elite microscope (Applied Precision) with a 100× N.A. 1.4 oil-immersion objective. Image deconvolution, projection, and colorization were performed using the SoftWoRx package and Photoshop CC 2014 (Adobe).

### Western blotting

For anti-GFP western blots (Fig. 2A), 20-30 adult worms of the indicated genotypes were picked into SDS sample buffer and lysed by boiling for 30 min, with occasional vortexing. Whole-worm lysates were separated on 4-12% polyacrylamide gradient gels and blotted onto nitrocellulose membranes. Antibodies against GFP (Roche, #11814460001) and α-tubulin (Sigma-Aldrich, #05-829 EMD MILLIPORE) were used at 1:1000 and 1:5000 respectively. HRP-conjugated secondary antibodies (Jackson Laboratory, #115-035-068) and ECL reagents (Amersham) were used for detection.

To quantify western blots, TIF images were recorded for each blot using a Chemidoc system (Bio-Rad), converted to 8-bit grayscale using Adobe Photoshop, and the integrated intensity of each GFP and α-tubulin band was calculated using ImageJ. The GFP band intensity was normalized by dividing by the corresponding α-tubulin band intensity. Each normalized GFP band intensity was expressed as a percentage of the intensity at t=0.

For anti-FLAG western blots (Fig. 3B), a synchronized dauer culture was generated as previously described (Wang and Kim, 2003). Animals were released from dauer by feeding with HB101 and cultured for six hours at 25°C with 150 rpm shaking. A ‘0 minute’ sample (1500 animals) was taken, and then either 0.25% ethanol or 1 mM auxin was added. At the indicated time points, 1500 animals were harvested, washed, and resuspended in 100 µl of M9+gelatin. After addition of 30 µl of 4× SDS sample buffer, lysates were made by boiling for 10 min, freezing on dry ice for 20 min, and boiling for 10 min. Proteins were resolved, probed and imaged as described previously (Ward, 2015). Anti-FLAG conjugated to horseradish peroxidase (1:2000, Sigma, #8592) was used, and the blot was developed using SuperSignal West Femto ECL substrate (Thermo Scientific, #34095).
